# An ortho­rhom­bic polymorph of 2-(1,3-benzothia­zol-2-yl)-6-eth­oxy­phenol

**DOI:** 10.1107/S1600536812033879

**Published:** 2012-08-04

**Authors:** Hadi Kargar, Reza Kia, Zahra Sharafi, Hossein Jalali Jahromi, Muhammad Nawaz Tahir

**Affiliations:** a Department of Chemistry, Payame Noor University, PO Box 19395-3697 Tehran, I. R. of IRAN; b Department of Chemistry, Science and Research Branch, Islamic Azad University, Tehran, Iran; c Structural Dynamics of (Bio)Chemical Systems, Max Planck Institute for Biophysical Chemistry, Am Fassberg 11, 37077 Göttingen, Germany; d Department of Chemistry, Marvdasht Branch, Islamic Azad University, Marvdasht, Iran; e Department of Chemistry and Chemical Engineering, Mahshahr Branch, Islamic Azad University, Mahshahr, Iran; f Department of Physics, University of Sargodha, Punjab, Pakistan

## Abstract

In the title mol­ecule, C_15_H_13_NO_2_S, an intra­molecular O—H⋯N hydrogen bond forms an *S*(6) ring motif. The benzothia­zole ring system and the benzene ring form a dihedral angle of 8.9 (3) Å. In the crystal, mol­ecules are linked by weak C—H⋯O hydrogen bonds, forming chains along the *b* axis. In addition, π–π inter­actions [centroid–centroid distances = 3.772 (4) and 3.879 (4) Å] are observed.

## Related literature

For the monoclinic polymorph, see: Lakshmanan *et al.* (2011 ▶). For background to and examples of the structures of Schiff base ligands see: Kargar *et al.* (2011 ▶); Kia *et al.* (2010 ▶). For hydrogen-bond motifs, see: Bernstein *et al.* (1995 ▶). For standard bond lengths, see: Allen *et al.* (1987 ▶).
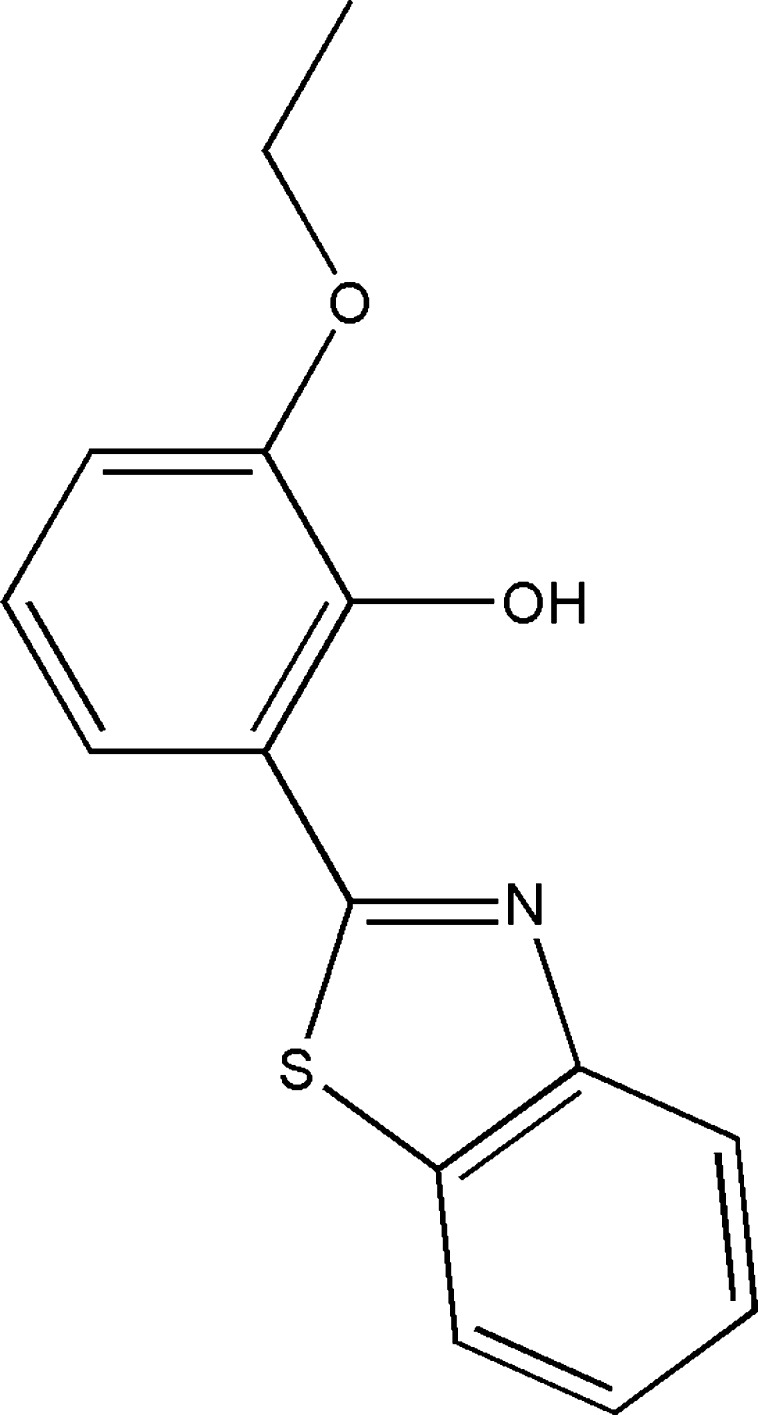


## Experimental

### 

#### Crystal data

C_15_H_13_NO_2_S
*M* *_r_* = 271.32
Orthorhombic, 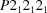

*a* = 4.8728 (10) Å
*b* = 11.711 (3) Å
*c* = 23.378 (6) Å
*V* = 1334.1 (6) Å^3^
*Z* = 4
Mo *K*α radiation
μ = 0.24 mm^−1^
*T* = 296 K
0.33 × 0.23 × 0.21 mm

#### Data collection

Bruker SMART APEXII CCD diffractometer
Absorption correction: multi-scan (*SADABS*; Bruker, 2005 ▶) *T*_min_ = 0.925, *T*_max_ = 0.952
4083 measured reflections
2239 independent reflections
1207 reflections with *I* > 2σ(*I*)
*R*_int_ = 0.073

#### Refinement

*R*[*F* ^2^ > 2σ(*F* ^2^)] = 0.068
*wR*(*F* ^2^) = 0.144
*S* = 0.99
2239 reflections
173 parameters
H-atom parameters constrained
Δρ_max_ = 0.19 e Å^−3^
Δρ_min_ = −0.30 e Å^−3^
Absolute structure: Flack (1983 ▶), 874 Friedel pairs
Flack parameter: −0.1 (2)

### 

Data collection: *APEX2* (Bruker, 2005 ▶); cell refinement: *SAINT* (Bruker, 2005 ▶); data reduction: *SAINT*; program(s) used to solve structure: *SHELXTL* (Sheldrick, 2008 ▶); program(s) used to refine structure: *SHELXTL*; molecular graphics: *SHELXTL*; software used to prepare material for publication: *SHELXTL* and *PLATON* (Spek, 2009 ▶).

## Supplementary Material

Crystal structure: contains datablock(s) global, I. DOI: 10.1107/S1600536812033879/lh5505sup1.cif

Structure factors: contains datablock(s) I. DOI: 10.1107/S1600536812033879/lh5505Isup2.hkl

Supplementary material file. DOI: 10.1107/S1600536812033879/lh5505Isup3.cml

Additional supplementary materials:  crystallographic information; 3D view; checkCIF report

## Figures and Tables

**Table 1:** Hydrogen-bond geometry (Å, °)

*D*—H⋯*A*	*D*—H	H⋯*A*	*D*⋯*A*	*D*—H⋯*A*
O1—H1⋯N1	0.88	1.76	2.592 (6)	158
C2—H2⋯O1^i^	0.93	2.55	3.372 (8)	148

Symmetry code: (i) 
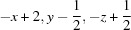
.

**Fig. 1.**
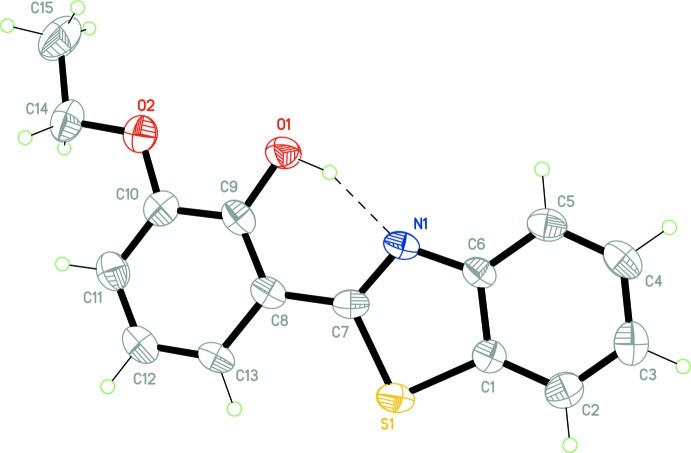
The molecular structure of the title compound, showing 40% probability displacement ellipsoids. The dashed line indicates an intramolecular hydrogen bond.

**Fig. 2.**
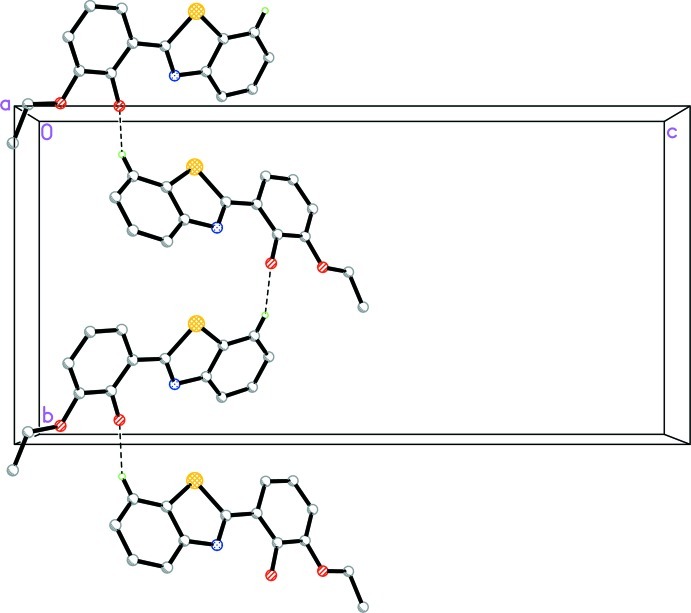
Part of the crystal structure with weak hydrogen bonds shown as dashed lines.

## supplementary crystallographic information

### Comment

In continuation of our work on the crystal structures of Schiff base ligands from different substituted salicylaldehyde and amines (Kargar *et al.,* 2011; Kia *et al.,* 2010), we have determined the X-ray structure of the title compound. The crystal structure of a monoclinic polymorph of the title compound has already been published (Lakshmanan *et al.*, 2011).

The molecular structure of the title compound is shown in Fig. 1. The bond lengths (Allen *et al.,* 1987) and angles are within the normal ranges. An intramolecular O—H···N hydrogen bond forms a *S(6)* ring motif (Bernstein *et al.,* 1995). In the crystal, molecules are linked by weak intermolecular C—H···O hydrogen bonds forming one-dimensional chains along the *b* axis (Table 2, Fig. 2). In addition, weak intermolecular π–π interactions are observed [*Cg*1···*Cg*2^ii^ = 3.772 (4) Å, (ii) -1 + x, y, z; *Cg*1···*Cg*3^iii^ = 3.879 (4), (iii) 1 + x, y, z, *Cg*1, *Cg*2 and *Cg*3 are centroid of S1/C1/C6/N1/C7, C1–C6, and C8–C13 rings].

### Experimental

The title compound was synthesized by adding 3-ethoxysalicylaldehyde (2 mmol) to a solution of 2-aminothiophenol (2mmol) in ethanol (30 ml). The mixture was refluxed with stirring for half an hour. The resultant solution was filtered. Light-yellow prismatic single crystals of the title compound suitable for X-ray structure determination were recrystallized from ethanol by slow evaporation of the solvent at room temperature over several days.

### Refinement

The O-bound H atom was located in a difference Fourier map and constrained to refine on the parent atom with U_iso_(H) = 1.5U_eq_(O). The other H-atoms were included in calculated positions and treated as riding atoms: C—H = 0.93, 0.97 and 0.96 Å for CH, CH_2_ and CH_3_ H-atoms, respectively, with U_iso_ (H) = k × U_eq_(C), k = 1.2 for CH, CH_2_ and 1.5 for CH_3_.

### Crystal data

**Table:**

C_15_H_13_NO_2_S	*F*(000) = 568
*M**_r_* = 271.32	*D*_x_ = 1.351 Mg m^−^^3^
Orthorhombic, *P*2_1_2_1_2_1_	Mo *K*α radiation, λ = 0.71073 Å
Hall symbol: P 2ac 2ab	Cell parameters from 864 reflections
*a* = 4.8728 (10) Å	θ = 2.5–28.8°
*b* = 11.711 (3) Å	µ = 0.24 mm^−^^1^
*c* = 23.378 (6) Å	*T* = 296 K
*V* = 1334.1 (6) Å^3^	Prism, light-yellow
*Z* = 4	0.33 × 0.23 × 0.21 mm

### Data collection

**Table:**

Bruker SMART APEXII CCD diffractometer	2239 independent reflections
Radiation source: fine-focus sealed tube	1207 reflections with *I* > 2σ(*I*)
Graphite monochromator	*R*_int_ = 0.073
ω scans	θ_max_ = 25.0°, θ_min_ = 1.7°
Absorption correction: multi-scan (*SADABS*; Bruker, 2005)	*h* = −3→5
*T*_min_ = 0.925, *T*_max_ = 0.952	*k* = −9→13
4083 measured reflections	*l* = −27→19

### Refinement

**Table:**

Refinement on *F*^2^	Secondary atom site location: difference Fourier map
Least-squares matrix: full	Hydrogen site location: inferred from neighbouring sites
*R*[*F*^2^ > 2σ(*F*^2^)] = 0.068	H-atom parameters constrained
*wR*(*F*^2^) = 0.144	*w* = 1/[σ^2^(*F*_o_^2^) + (0.0413*P*)^2^] where *P* = (*F*_o_^2^ + 2*F*_c_^2^)/3
*S* = 0.99	(Δ/σ)_max_ < 0.001
2239 reflections	Δρ_max_ = 0.19 e Å^−^^3^
173 parameters	Δρ_min_ = −0.30 e Å^−^^3^
0 restraints	Absolute structure: Flack (1983), 874 Friedel pairs
Primary atom site location: structure-invariant direct methods	Flack parameter: −0.1 (2)

### Special details

**Table:**

Geometry. All esds (except the esd in the dihedral angle between two l.s. planes) are estimated using the full covariance matrix. The cell esds are taken into account individually in the estimation of esds in distances, angles and torsion angles; correlations between esds in cell parameters are only used when they are defined by crystal symmetry. An approximate (isotropic) treatment of cell esds is used for estimating esds involving l.s. planes.
Refinement. Refinement of F^2^ against ALL reflections. The weighted R-factor wR and goodness of fit S are based on F^2^, conventional R-factors R are based on F, with F set to zero for negative F^2^. The threshold expression of F^2^ > 2sigma(F^2^) is used only for calculating R-factors(gt) etc. and is not relevant to the choice of reflections for refinement. R-factors based on F^2^ are statistically about twice as large as those based on F, and R- factors based on ALL data will be even larger.

### Fractional atomic coordinates and isotropic or equivalent isotropic displacement parameters (Å^2^)

**Table:**

	*x*	*y*	*z*	*U*_iso_*/*U*_eq_	
C1	1.2770 (11)	0.2177 (4)	0.2096 (3)	0.0485 (16)	
C2	1.4128 (13)	0.1882 (5)	0.1606 (3)	0.0647 (19)	
H2	1.3725	0.1196	0.1423	0.078*	
C3	1.6064 (14)	0.2590 (6)	0.1386 (3)	0.073 (2)	
H3	1.6970	0.2397	0.1049	0.087*	
C4	1.6692 (13)	0.3614 (6)	0.1667 (3)	0.0670 (18)	
H4	1.8030	0.4093	0.1516	0.080*	
C5	1.5378 (14)	0.3923 (5)	0.2160 (3)	0.0599 (17)	
H5	1.5816	0.4606	0.2342	0.072*	
C6	1.3392 (11)	0.3211 (5)	0.2386 (3)	0.0458 (15)	
N1	1.1883 (9)	0.3431 (4)	0.2872 (2)	0.0468 (12)	
C8	0.8322 (12)	0.2602 (5)	0.3462 (3)	0.0509 (16)	
C9	0.8043 (12)	0.3576 (5)	0.3797 (3)	0.0530 (16)	
C10	0.6091 (12)	0.3608 (6)	0.4232 (3)	0.0603 (17)	
C11	0.4481 (14)	0.2664 (6)	0.4350 (3)	0.0664 (19)	
H11	0.3218	0.2685	0.4648	0.080*	
C12	0.4756 (15)	0.1698 (6)	0.4026 (3)	0.0686 (18)	
H12	0.3661	0.1067	0.4105	0.082*	
C13	0.6598 (12)	0.1642 (5)	0.3590 (3)	0.0568 (16)	
H13	0.6740	0.0979	0.3373	0.068*	
C14	0.3939 (15)	0.4754 (6)	0.4949 (3)	0.084 (2)	
H14A	0.4162	0.4185	0.5246	0.101*	
H14B	0.2133	0.4663	0.4780	0.101*	
C15	0.4237 (18)	0.5927 (7)	0.5194 (3)	0.118 (3)	
H15A	0.6144	0.6088	0.5257	0.176*	
H15B	0.3265	0.5970	0.5551	0.176*	
H15C	0.3492	0.6476	0.4932	0.176*	
C7	1.0189 (12)	0.2585 (4)	0.2991 (2)	0.0472 (15)	
O1	0.9557 (8)	0.4529 (3)	0.37058 (16)	0.0633 (12)	
H1	1.0595	0.4317	0.3417	0.095*	
O2	0.5994 (9)	0.4617 (4)	0.45248 (18)	0.0741 (13)	
S1	1.0285 (3)	0.14638 (12)	0.24914 (8)	0.0574 (5)	

### Atomic displacement parameters (Å^2^)

**Table:**

	*U*^11^	*U*^22^	*U*^33^	*U*^12^	*U*^13^	*U*^23^
C1	0.039 (4)	0.049 (4)	0.057 (4)	0.007 (3)	−0.008 (3)	0.004 (3)
C2	0.064 (5)	0.063 (4)	0.067 (5)	0.008 (4)	−0.006 (4)	−0.003 (4)
C3	0.065 (5)	0.088 (6)	0.064 (5)	0.004 (4)	0.007 (4)	0.009 (5)
C4	0.064 (4)	0.063 (5)	0.074 (5)	−0.013 (4)	−0.008 (4)	0.019 (4)
C5	0.066 (5)	0.048 (4)	0.066 (4)	−0.001 (4)	−0.012 (4)	0.003 (4)
C6	0.038 (3)	0.036 (3)	0.063 (5)	0.005 (3)	−0.005 (3)	0.009 (3)
N1	0.040 (3)	0.036 (3)	0.064 (3)	−0.001 (3)	−0.010 (3)	0.001 (3)
C8	0.043 (4)	0.045 (4)	0.065 (4)	0.001 (3)	−0.010 (3)	0.008 (4)
C9	0.043 (4)	0.049 (4)	0.067 (5)	0.005 (3)	−0.006 (3)	0.018 (4)
C10	0.055 (4)	0.066 (4)	0.060 (4)	0.005 (4)	−0.006 (3)	−0.001 (4)
C11	0.055 (5)	0.076 (5)	0.068 (5)	−0.005 (4)	0.002 (4)	0.010 (4)
C12	0.063 (4)	0.065 (5)	0.078 (5)	−0.011 (4)	−0.004 (4)	0.019 (4)
C13	0.057 (4)	0.041 (4)	0.072 (5)	−0.006 (4)	−0.008 (4)	0.008 (4)
C14	0.083 (6)	0.105 (6)	0.063 (5)	0.004 (5)	0.014 (4)	−0.007 (5)
C15	0.142 (8)	0.116 (7)	0.095 (6)	0.016 (6)	0.027 (6)	−0.035 (5)
C7	0.046 (4)	0.038 (3)	0.057 (4)	0.008 (3)	−0.015 (3)	0.000 (3)
O1	0.066 (3)	0.045 (2)	0.078 (3)	0.000 (2)	0.011 (2)	−0.004 (2)
O2	0.073 (3)	0.077 (3)	0.072 (3)	−0.004 (3)	0.015 (3)	−0.007 (3)
S1	0.0528 (9)	0.0410 (7)	0.0784 (11)	−0.0014 (8)	−0.0047 (10)	−0.0030 (10)

### Geometric parameters (Å, º)

**Table:**

C1—C2	1.368 (8)	C9—C10	1.393 (7)
C1—C6	1.421 (7)	C10—O2	1.366 (7)
C1—S1	1.737 (6)	C10—C11	1.384 (8)
C2—C3	1.358 (8)	C11—C12	1.368 (8)
C2—H2	0.9300	C11—H11	0.9300
C3—C4	1.402 (8)	C12—C13	1.360 (8)
C3—H3	0.9300	C12—H12	0.9300
C4—C5	1.368 (8)	C13—H13	0.9300
C4—H4	0.9300	C14—O2	1.418 (7)
C5—C6	1.382 (7)	C14—C15	1.496 (9)
C5—H5	0.9300	C14—H14A	0.9700
C6—N1	1.377 (7)	C14—H14B	0.9700
N1—C7	1.318 (6)	C15—H15A	0.9600
C8—C9	1.391 (8)	C15—H15B	0.9600
C8—C7	1.428 (7)	C15—H15C	0.9600
C8—C13	1.435 (8)	C7—S1	1.758 (5)
C9—O1	1.355 (6)	O1—H1	0.8797
			
C2—C1—C6	120.8 (6)	C12—C11—C10	119.7 (6)
C2—C1—S1	131.4 (5)	C12—C11—H11	120.2
C6—C1—S1	107.7 (4)	C10—C11—H11	120.2
C3—C2—C1	120.0 (6)	C13—C12—C11	121.3 (6)
C3—C2—H2	120.0	C13—C12—H12	119.3
C1—C2—H2	120.0	C11—C12—H12	119.3
C2—C3—C4	119.7 (6)	C12—C13—C8	120.3 (6)
C2—C3—H3	120.1	C12—C13—H13	119.8
C4—C3—H3	120.1	C8—C13—H13	119.8
C5—C4—C3	121.4 (6)	O2—C14—C15	107.7 (6)
C5—C4—H4	119.3	O2—C14—H14A	110.2
C3—C4—H4	119.3	C15—C14—H14A	110.2
C4—C5—C6	119.4 (6)	O2—C14—H14B	110.2
C4—C5—H5	120.3	C15—C14—H14B	110.2
C6—C5—H5	120.3	H14A—C14—H14B	108.5
N1—C6—C5	125.2 (6)	C14—C15—H15A	109.5
N1—C6—C1	116.0 (5)	C14—C15—H15B	109.5
C5—C6—C1	118.7 (5)	H15A—C15—H15B	109.5
C7—N1—C6	111.6 (5)	C14—C15—H15C	109.5
C9—C8—C7	120.5 (5)	H15A—C15—H15C	109.5
C9—C8—C13	117.9 (6)	H15B—C15—H15C	109.5
C7—C8—C13	121.5 (6)	N1—C7—C8	123.4 (5)
O1—C9—C8	122.2 (5)	N1—C7—S1	113.9 (4)
O1—C9—C10	117.7 (6)	C8—C7—S1	122.6 (5)
C8—C9—C10	120.0 (6)	C9—O1—H1	101.7
O2—C10—C11	124.9 (6)	C10—O2—C14	118.2 (5)
O2—C10—C9	114.3 (6)	C1—S1—C7	90.7 (3)
C11—C10—C9	120.7 (6)		
			
C6—C1—C2—C3	−1.2 (8)	C8—C9—C10—C11	2.3 (9)
S1—C1—C2—C3	−178.3 (5)	O2—C10—C11—C12	179.8 (6)
C1—C2—C3—C4	1.0 (9)	C9—C10—C11—C12	−1.8 (9)
C2—C3—C4—C5	−0.5 (10)	C10—C11—C12—C13	0.4 (10)
C3—C4—C5—C6	0.1 (9)	C11—C12—C13—C8	0.5 (10)
C4—C5—C6—N1	−178.7 (5)	C9—C8—C13—C12	0.1 (9)
C4—C5—C6—C1	−0.3 (8)	C7—C8—C13—C12	−177.2 (6)
C2—C1—C6—N1	179.4 (5)	C6—N1—C7—C8	−178.1 (5)
S1—C1—C6—N1	−2.9 (6)	C6—N1—C7—S1	−1.6 (6)
C2—C1—C6—C5	0.8 (8)	C9—C8—C7—N1	5.5 (8)
S1—C1—C6—C5	178.6 (4)	C13—C8—C7—N1	−177.3 (5)
C5—C6—N1—C7	−178.6 (5)	C9—C8—C7—S1	−170.7 (4)
C1—C6—N1—C7	3.0 (6)	C13—C8—C7—S1	6.6 (8)
C7—C8—C9—O1	−1.9 (8)	C11—C10—O2—C14	−5.8 (9)
C13—C8—C9—O1	−179.3 (5)	C9—C10—O2—C14	175.7 (5)
C7—C8—C9—C10	175.9 (5)	C15—C14—O2—C10	−178.9 (6)
C13—C8—C9—C10	−1.5 (8)	C2—C1—S1—C7	179.0 (6)
O1—C9—C10—O2	−1.2 (8)	C6—C1—S1—C7	1.5 (4)
C8—C9—C10—O2	−179.1 (5)	N1—C7—S1—C1	0.0 (4)
O1—C9—C10—C11	−179.8 (5)	C8—C7—S1—C1	176.5 (5)

### Hydrogen-bond geometry (Å, º)

**Table:**

*D*—H···*A*	*D*—H	H···*A*	*D*···*A*	*D*—H···*A*
O1—H1···N1	0.88	1.76	2.592 (6)	158
C2—H2···O1^i^	0.93	2.55	3.372 (8)	148

Symmetry code: (i) −*x*+2, *y*−1/2, −*z*+1/2.
